# Hemorrhage From Left Hepatic Artery Pseudoaneurysm as a Complication of Acute Pancreatitis in a Patient With Fibromuscular Dysplasia

**DOI:** 10.14309/crj.0000000000001098

**Published:** 2023-07-10

**Authors:** Dennis Wang, Gordon Yip, David Anthony Szalay, Paul Moayyedi

**Affiliations:** 1 McMaster University Adult Gastroenterology Residency Program, Hamilton, Ontario, Canada; 2 Department of Radiology, McMaster University, Hamilton, Ontario, Canada; 3 Division of Vascular Surgery, Department of Surgery, McMaster University, Hamilton, Ontario, Canada; 4 Division of Gastroenterology, Department of Medicine, McMaster University, Hamilton, Ontario, Canada

**Keywords:** acute pancreatitis, fibromuscular dysplasia, pseudoaneurysm, embolization

## Abstract

Hepatic artery pseudoaneurysms are a rare complication of pancreatitis, and their rupture and bleeding cause high mortality. We present the case of a 76-year-old woman with fibromuscular dysplasia who developed a new left hepatic artery pseudoaneurysm within a week of her first episode of acute pancreatitis and later suffered an acute pseudoaneurysm bleed successfully treated with transcatheter coil embolization. To the best of our knowledge, this is the first case reported of a patient with fibromuscular dysplasia with pancreatitis-related pseudoaneurysm formation. One must consider pseudoaneurysms and associated bleeding as complications of acute pancreatitis because prompt recognition can lead to timely management.

## INTRODUCTION

Around 20% of patients with acute pancreatitis develop moderate to severe disease, with complications including peripancreatic fluid collections, pseudocysts, and multiorgan failure.^1^ Arterial complications, including pseudoaneurysm development, accounts only for 4%–10% of these complications.^2^ Pseudoaneurysms differ from normal aneurysms in that the wall consists of fibrous tissue rather than the normal 3 vessel layers of tunica intima, media, and adventitia. The commonest artery to be involved in a pseudoaneurysm is the splenic artery because it is most anatomically connected to the pancreas. Only 8%–19% of pseudoaneurysms from pancreatitis arise from the hepatic artery, and they typically involve the common hepatic artery (CHA) and not its branches.^3–5^ We report this case of a 76-year-old woman with fibromuscular dysplasia (FMD) who developed a new left hepatic artery pseudoaneurysm (HAP) within a week of her first episode of acute pancreatitis and later developed an acute pseudoaneurysm hemorrhage requiring transcatheter coil embolization.

## CASE REPORT

A 76-year-old woman with a medical history of FMD involving the bilateral renal arteries, splenic arterial branches, and the distal superior mesenteric artery was admitted for 6 days for acute pancreatitis presenting as acute epigastric pain and vomiting. She never had any previous pancreaticobiliary issues, and there were no notable triggers for this episode such as new medications, alcohol use, or hypercalcemia. On admission, her lipase was 915 U/L and hemoglobin was 136 g/L. A computed tomography (CT) scan on admission demonstrated ill-defined peripancreatic inflammatory stranding, and an abdominal ultrasound showed gallbladder sludge without gallstones. She received intravenous fluid resuscitation and analgesia and was eventually discharged. Hemoglobin at discharge was 82 g/L.

**Figure 1.**
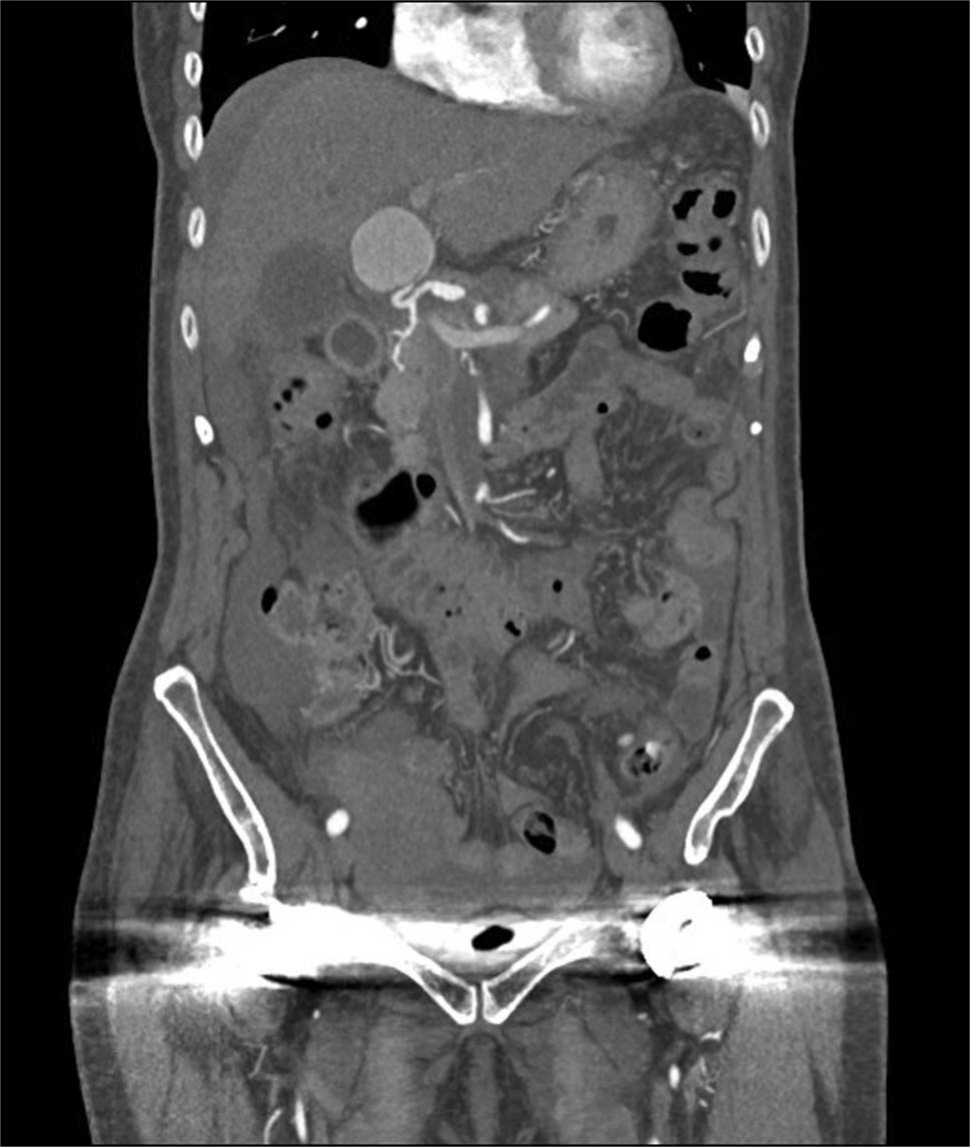
Coronal view of pre-embolization abdominal computed tomography with left hepatic artery pseudoaneurysm.

**Figure 2.**
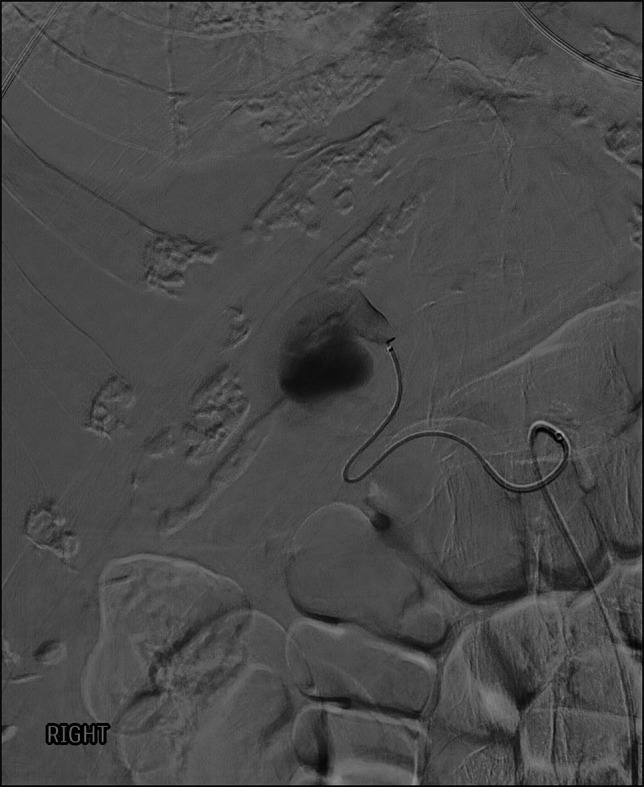
Pre-embolization angiogram of left hepatic artery pseudoaneurysm.

**Figure 3.**
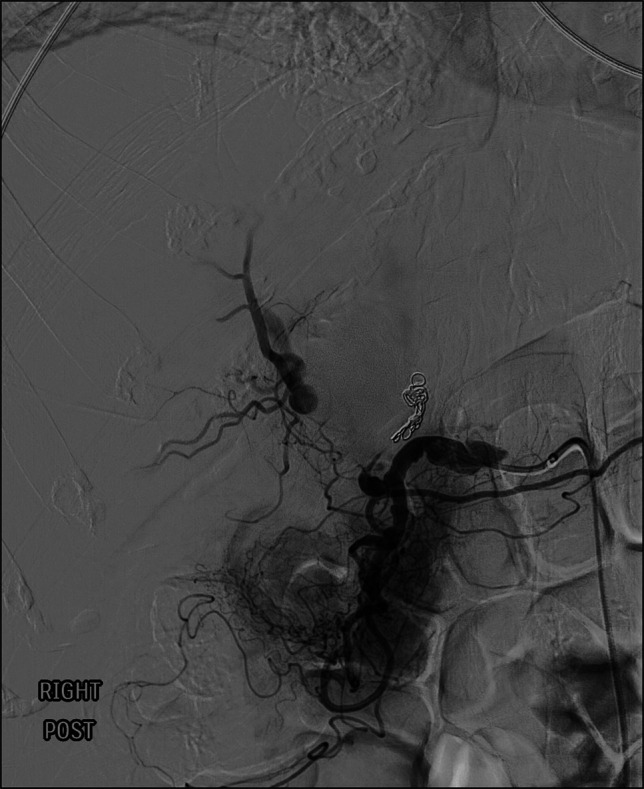
Postembolization angiogram of left hepatic artery pseudoaneurysm, with coils in place.

Two days later, she presented to the emergency department of a community hospital with acute epigastric pain and syncope. Her hemoglobin was 67 g/L. Abdominal CT demonstrated a suspected gastroduodenal artery pseudoaneurysm with associated hemoperitoneum. She was transferred to the intensive care unit of a tertiary medical center. On arrival, a CT angiogram (CTA) demonstrated a 35-mm pseudoaneurysm arising from the left hepatic artery, adjacent to a 36 × 27 × 21-mm pancreatic pseudocyst (Figure 1). The CTA also showed multiple other pseudocysts. The interventional radiology service was urgently consulted for transcatheter embolization. The right common femoral artery was cannulated using ultrasound guidance, and the CHA was accessed with a catheter and an angled glidewire. The left HAP was identified with angiography from the mid CHA. Two interlock helical fibered coils and 2 interlock coils were deployed into the pseudoaneurysm with successfully embolization (Figures 2 and 3). During embolization, aneurysmal change involving the CHA, gastroduodenal artery, and right hepatic arteries was seen. The rheumatology service reviewed her case and determined that there was no active vasculitis involved based on unremarkable inflammatory markers and autoimmune serology. An abdominal ultrasound 2 days after embolization showed thrombosis of the pseudoaneurysm. She was discharged on the fifth day of admission.

**Figure 4.**
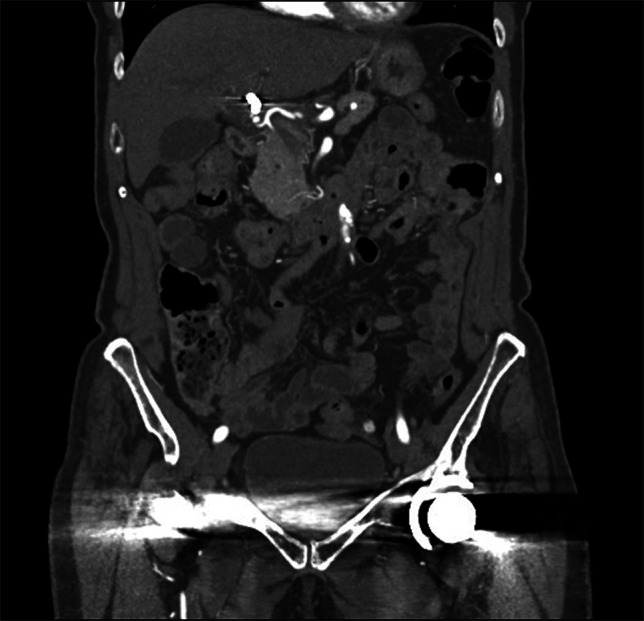
Coronal view of postembolization abdominal computed tomography with left hepatic artery pseudoaneurysm, with coils in place.

For 2 weeks after discharge, the patient was deconditioned. However, she gradually resumed ambulation. Since then, she has had recurrent episodes of mild upper abdominal discomfort. An outpatient CTA 2 months later showed embolization of the left HAP, as well as a decreased size of the pancreatic pseudocysts (Figure 4). She has not had any more episodes of bleeding over 4 months. She is being followed as an outpatient with imaging surveillance, with the next CT scan scheduled for 6 months after her initial presentation.

## DISCUSSION

The etiology of HAP is varied and includes acute or chronic pancreatitis as well as physical trauma, iatrogenic injury from hepatobiliary surgeries, vasculitides such as Behcet disease, and other vascular conditions such as cystic medial necrosis.^6–9^ The pathophysiology of pseudoaneurysm formation in pancreatitis is believed to involve vessel wall erosion by enzyme-rich pancreatic fluid released during inflammation.^9,10^ This is supported by case reports in which HAPs are found near peripancreatic fluid collections or pseudocysts.^5,11^ Necrotizing vasculitis from pancreatitis may also promote pseudoaneurysm formation.^6^

FMD is an idiopathic, nonatherosclerotic arterial condition that results in arterial stenosis and aneurysm formation.^12^ As we describe the first known case of pancreatitis-related HAP hemorrhage in patients with FMD, this may suggest that pancreatitis-related pseudoaneurysm formation may not be as common in this group, compared with patients with other vascular conditions.

Pseudoaneurysms typically form 3–5 weeks after the onset of acute pancreatitis.^3^ Patients can either present asymptomatically or with abdominal pain, jaundice, or symptoms of hemorrhage such as syncope.^13^ HAP rupture occurs in up to 76% of patients, with a mortality rate of 90% that decreases to 15%–50% with treatment.^2,7^ Rarely, HAPs may spontaneously thrombose.^7,10^

Multisection CT angiography is the best initial test to detect pseudoaneurysms or bleeding related to pancreatitis, with a sensitivity and specificity of 94% and 90%, respectively.^14^ Ultrasound and CT are less sensitive than CTA, but may diagnose pseudoaneurysms more quickly to facilitate treatment.^7,9^ First-line treatment for HAP is angiography with transcatheter embolization because it has less procedural risk and can more precisely locate and treat lesions compared with surgery.^13^ Although we present only one case of a patient with FMD with successful HAP embolization, this may suggest that patients with FMD can be managed similarly to the general population. Other treatment methods described in literature include endovascular deployment of a covered stent, endovascular glue embolization, and direct percutaneous puncture and injection of an embolic agent under ultrasound guidance.^8,9,13,15^ If embolization or endovascular strategies fail, then surgical management such as vessel ligation or partial pancreatectomy may be required.^2^

HAPs must be considered as a potential complication of acute pancreatitis because the pseudoaneurysms can rupture and have a high mortality if left untreated. Early recognition, along with timely diagnostic investigations and prompt referral to interventional radiology or surgery, is essential for the prevention or treatment of hemorrhage from pancreatitis-related pseudoaneurysms.

## DISCLOSURES

Author contributions: D. Wang: conceived of case report, obtained written consent from patient, reviewed literature, wrote the manuscript, and provided final approval of the manuscript. G. Yip: provided radiographic images, reviewed literature, revised manuscript for important intellectual content, and provided final approval of the manuscript. D.A. Szalay: reviewed literature, revised manuscript for important intellectual content, and provided final approval of the manuscript. P. Moayyedi: revised manuscript for important intellectual content and provided final approval of the manuscript. D. Wang is the article guarantor.

Financial disclosure: None to report.

Informed consent was obtained for this case report.
